# Unveiling radial breathing mode in a particle-on-mirror plasmonic nanocavity

**DOI:** 10.1515/nanoph-2021-0506

**Published:** 2022-01-04

**Authors:** Qifa Wang, Chenyang Li, Liping Hou, Hanmou Zhang, Xuetao Gan, Kaihui Liu, Malin Premaratne, Fajun Xiao, Jianlin Zhao

**Affiliations:** Key Laboratory of Light Field Manipulation and Information Acquisition, Ministry of Industry and Information Technology, and Shaanxi Key Laboratory of Optical Information Technology, School of Physical Science and Technology, Northwestern Polytechnical University, Xi’an 710129, China; State Key Laboratory for Mesoscopic Physics, Collaborative Innovation Centre of Quantum Matter, School of Physics, Peking University, Beijing 100871, China; Advanced Computing and Simulation Laboratory (AχL), Department of Electrical and Computer Systems Engineering, Monash University, Clayton, Victoria 3800, Australia

**Keywords:** dark mode, nanoparticles, plasmonic nanocavity, radial breathing mode

## Abstract

Plasmonic radial breathing mode (RBM), featured with radially oscillating charge density, arises from the surface plasmon waves confined in the flat nanoparticles. The zero net dipole moment endows the RBM with an extremely low radiation yet a remarkable intense local field. On the other hand, owing to the dark mode nature, the RBMs routinely escape from the optical measurements, severely preventing their applications in optoelectronics and nanophotonics. Here, we experimentally demonstrate the existence of RBM in a hexagonal Au nanoplate-on-mirror nanocavity using a far-field linear-polarized light source. The polarization-resolved scattering measurements cooperated with the full-wave simulations elucidate that the RBM originates from the standing plasmon waves residing in the Au nanoplate. Further numerical analysis shows the RBM possesses the remarkable capability of local field enhancement over the other dark modes in the same nanocavity. Moreover, the RBM is sensitive to the gap and nanoplate size of the nanocavity, providing a straightforward way to tailor the wavelength of RBM from the visible to near-infrared region. Our approach provides a facile optical path to access to the plasmonic RBMs and may open up a new route to explore the intriguing applications of RBM, including surface-enhanced Raman scattering, enhanced nonlinear effects, nanolasers, biological and chemical sensing.

## 1 Introduction

Metallic nanoparticles with localized plasmons modes [1, 2] could provide a unique way to squeeze light into nanoscale volume and profoundly enhance the light field near the particles. Generally, these plasmonic modes fall into two categories, i.e., the bright and the dark modes. The bright mode, such as the electric dipole, has a nonzero dipole moment. Therefore, it can be easily excited through optical means and reradiate considerable electromagnetic energy to the far-field. On the contrary, the dark mode [3] exemplified by the electric quadrupole, has a vanishing dipole moment. Consequently, it hardly couples with light, but can store electromagnetic energy more efficiently than the bright mode [4]. The resulting virtues of high local field enhancement and narrow spectral linewidth make the dark mode attractive for a plethora of applications, including surface-enhanced spectroscopy [5, 6], enhanced optical nonlinearity [7], nanolaser [8], biological and chemical sensing [9, 10].

Among the dark modes, the plasmonic radial breathing mode (RBM) is receiving a growing interest [11], [12], [13], [14], [15]. The RBM was first discovered in nuclear physics as a giant monopole resonance associated with the periodic compression and decompression of nuclei [16]. Now, the RBM is a ubiquitous phenomenon in the field of molecular [17], [18], [19], solid-state [20, 21] and acoustic physics [22]. In plasmonics, the RBM, featured with radially symmetric surface charge density oscillation, has been demonstrated in a variety of metallic structures such as nanodisks [11, 12, 14], nanosquares [23], nanotriangles [13], nanoapertures [15] and nanoclusters [24]. Due to the dark mode nature, the plasmonic breathing mode is mainly characterized using the electron beam, for instance, the cathodoluminescence [14, 25] and the scanning transmission electron microscopes equipped with the electron energy loss spectroscopy [3, 11, 14, 26]. These approaches provide unambiguous spectral analysis of the RBM with the ability to image the mode profile with unprecedented spatial resolution. However, for practical applications, such as optical sensing and surface-enhanced Raman scattering, it is indispensable to access to the RBM by non-invasive optical means with the ambient operating conditions. Unfortunately, optical probing of the RBM remains scarce, except for one uses a configuration combing the oblique illumination and the retardation effect [12].

Notably, the recently emerging nanoparticle-on-mirror structures [27], [28], [29], [30], [31], [32], [33], [34] serve as an ideal platform to explore the plasmonic dark modes including the anapole mode [34], the bonding dipole [35] and quadruple modes [32]. These plasmonic structures much resemble the metal–insulator–metal (MIM) waveguides [36], [37], [38], [39]. In this sense, the surface plasmon polaritons (SPPs) residing in the waveguide possess a much larger wave-vector than that of a photon in free space, therefore, greatly facilitate the excitation of plasmonic dark modes. Moreover, the MIM configuration affords great flexibility to tailor the optical responses through the structural parameter designing [38, 39]. Here, we report for the first time on the excitation of plasmonic RBM in hexagonal Au nanoplate-on-mirror (NPoM) structure using a linear polarized far-field light source. The combination of experimental data with simulation results and theoretical models reveals that the RBM holds intriguing characters of strong electric field enhancement and highly tunable dispersion. Our results shed light on the physics of plasmonic RBM and pave the way for its applications in optical sensing, surface-enhanced Raman scattering and enhanced optical nonlinearity.

## 2 Experimental section

### 2.1 Sample preparation

The hexagonal Au NPoM samples were fabricated using the bottom-up following with the self-assembly techniques [40]. First, a 50 nm Au film was deposited onto a silicon substrate using thermal evaporation at a rate of 2 Å/s. This Au film was transferred onto a SiO_2_/Si substrate via a template-stripping method [41], yielding an ultrasmooth Au surface with the root-mean-square surface roughness of 0.26 nm. Then, an Al_2_O_3_ spacer layer with thickness varying from 1–15 nm was deposited on the Au film using the atomic layer deposition at 90 °C with 1.13 Å/cycle. The thickness of the deposited Al_2_O_3_ spacer is linear with the number of reaction cycle, therefore, was well controlled through the cycle numbers. Finally, a diluted Au hexagonal nanoplates solution (Nanoseedz, Inc.) was drop-coated on the Al_2_O_3_ spacer layer to form the NPoM geometries.

### 2.2 Optical measurements

The dark-field scattering spectroscopy was performed using a home-built dark-field confocal microscope [42]. A polarization-controlled white light from a halogen lamp illuminated the sample through a 50× objective (Mitutoyo, NA 0.55) at an incident angle of 70°. The scattering light was collected with an upright 50× objective (Nikon NA 0.4), then was recorded as the dark image by a CCD camera. Meanwhile, after passing through a spatial filter, the scattering signal from the individual hexagonal Au NPoM was delivered to an imaging spectrometer (Andor Shamrock SR-303i) for spectral analysis.

### 2.3 Numerical simulations

The plasmonic responses of Au hexagonal NPoMs were calculated using the finite element method. The geometrical configurations of the hexagonal Au NPoMs were modeled as their experimental counterparts. Moreover, to match with the experimental samples, the corners and edges of the nanoplates were rounded by an 8 nm curvature. The permittivity of Au was taken from the experimental data of Johnson and Christy [43]. The refractive indices of the Al_2_O_3_ layer and cetyltrimethylammonium bromide (CTAB) surfactant polymer were taken as a constant of 1.55. The scattering fields of NPoMs were obtained through a two-step method, where the background field, derived from the plane wave incident on the substrate without the hexagonal Au nanoplate, was used as the excitation with the nanoplate present. To calculate the scattering spectra, the scattered field at each wavelength were collected within a cone of half-angle 23.6°, corresponding to the numerical aperture of the objective. The charge density of the plasmonic mode was derived by calculating the difference of the normal component of the electric field above and below the metal surface according to Gauss’s law.

## 3 Results and discussion

**Figure 1:**
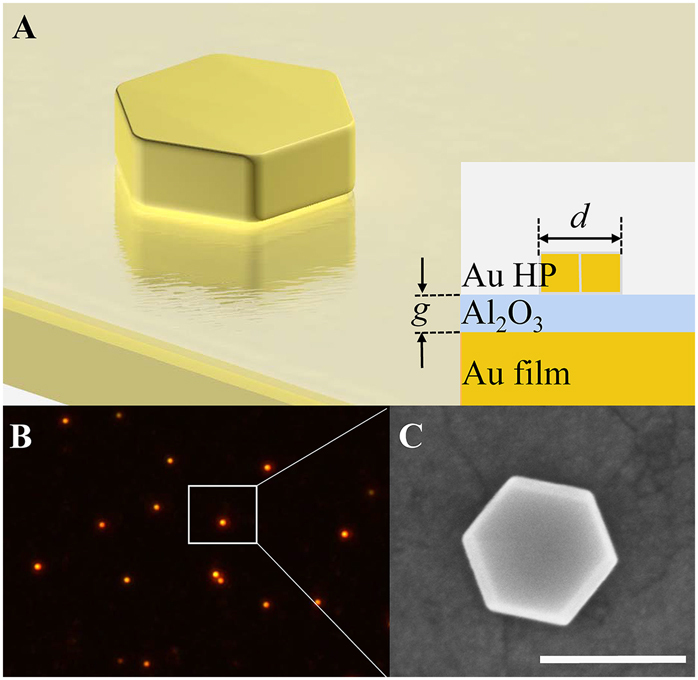
(A) Schematic of hexagonal Au nanoplate-on-mirror (NPoM) consisting a hexagonal Au nanoplate, an ultrasmooth Au film and an Al_2_O_3_ spacer. Inset shows the cross-section diagram of hexagonal Au NPoM. (B) Dark-field image of the hexagonal Au NPoM. The white box indicates a typical hexagonal Au nanoplate measured in our experiment. (C) SEM image of the hexagonal Au NPoM. The scale bar is 200 nm.

Figure 1A schematically shows the hexagonal Au NPoM sample under consideration, which is composed of a single hexagonal Au nanoplate and an ultrasmooth Au film spaced by an Al_2_O_3_ layer with the thickness of *g*. The hexagonal Au nanoplate is coated with a 2 nm CTAB and has an average thickness of 50 nm (see Supplementary Material, Figure S1). The size of the nanoplate is defined by the distance between two parallel edges *d*. Figure 1B displays the dark-field image of a representative hexagonal Au NPoM. As can be seen, the separations between hexagonal Au nanoplates are deliberately controlled over 8 μm to ensure the single-particle level scattering measurement. Figure 1C shows the scanning electronic microscope (SEM) image of the hexagonal Au NPoM, which exhibits a nearly perfect hexagonal shape with the particle size determined to be 180 nm.

**Figure 2:**
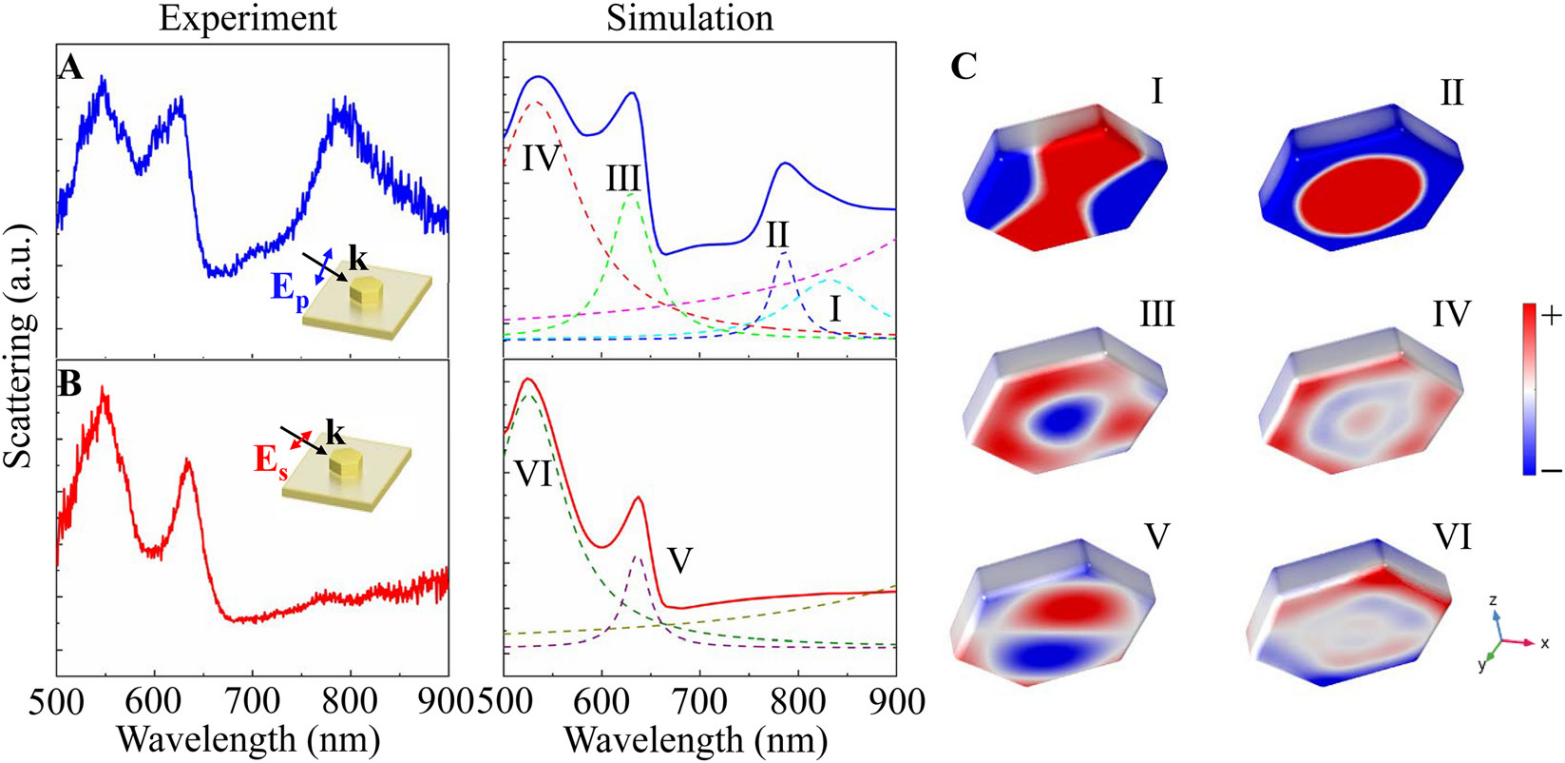
Experimental (left column) and simulated (right column) scattering spectra of hexagonal Au NPoM (*d* = 185 nm, *g* = 5 nm) illuminated by (A) p-polarized and (B) s-polarized beams. (C) Charge density distributions of modes I–VI with spectral positions labeled in (A) and (B).

The scattering measurements were carried out on the single hexagonal Au NPoM using a polarization-resolved micro-spectroscopy system. The left panels of Figure 2A and B shows the experimental scattering spectra of a hexagonal Au NPoM (*d* = 185 nm, *g* = 5 nm) under the excitation of p- and s-polarized beams, respectively. Due to the strong interaction between the Au nanoplate and the underlying metal film, a wealth of spectral peaks appears compared with the scenario of the bare nanoplate sitting on a silica substrate (see Supplementary Material, Figure S2A). To uncover the origins of these spectral peaks, we calculate the scattering spectra of the hexagonal Au NPoM with a full-wave simulation based on the finite element method (FEM) as shown in the right panels of Figure 2A and B. The excellent agreement between our measured and simulated spectra allows us to decompose the spectra with multiple Lorentzian functions according to the charge density maps of the NPoM. In the following, for briefness, we constraint the analyses on the mode types of the Au nanoplate, though their imaged modes are produced in the Au film at meanwhile. Under the p-polarized illumination, the broad spectral peak at the longer wavelength can be interpreted as the superposition of a quadruple mode (mode I, *λ* = 832 nm) and a fundamental RBM (mode II, *λ* = 787 nm), as confirmed by the charge density maps in Figure 2C. As seen, the RBM features a circular charge nodal line and holds considerable charge around the center of the nanoplate. This unique charge distribution is distinguished from conventional plasmonic modes, such as electric dipole mode, for which the charge is localized at the periphery of the structure. Of note is that the Au nanoplate, the Al_2_O_3_ spacer together with the ultrasmooth Au film equivalently forms a metal–insulator–metal (MIM) waveguide. In this sense, the SPPs mode of this MIM waveguide can be readily excited by the p-polarized (TM) beam in the present of the subwavelength nanoplate. The in-plane propagating SPPs carry a large wave-vector and considerable phase retardation, making the RBM attainable.

As truncated by the bottom edges of nanoplate, the SPPs are reflected back and forth within the MIM waveguide and eventually escape to free space, rendering the breathing mode visible. In addition, two peaks in the shorter wavelength region are identified as the second- (mode III, *λ* = 630 nm) and third-order RBMs (mode IV, *λ* = 533 nm), as the evidence from the number of circular nodal lines in Figure 2C. Obvious distortions are also found in these charge maps due to the more complex phase retardation at the short wavelength. For the s-polarized illumination, two profound peaks are observed and are denoted as the modes V and VI. These two modes can be assigned with mode indices of (1, 1) and (1, 3), according to their charge pattern shown in Figure 2C. Since these two modes are odd parity, they can be excited directly by the s-polarized beam even at normal incidence (see Supplementary Material, Figure S2B). In particular, the absence of a spectral peak at the longer wavelength further confirms the RBM arises from the SPPs of the MIM waveguide.

**Figure 3:**
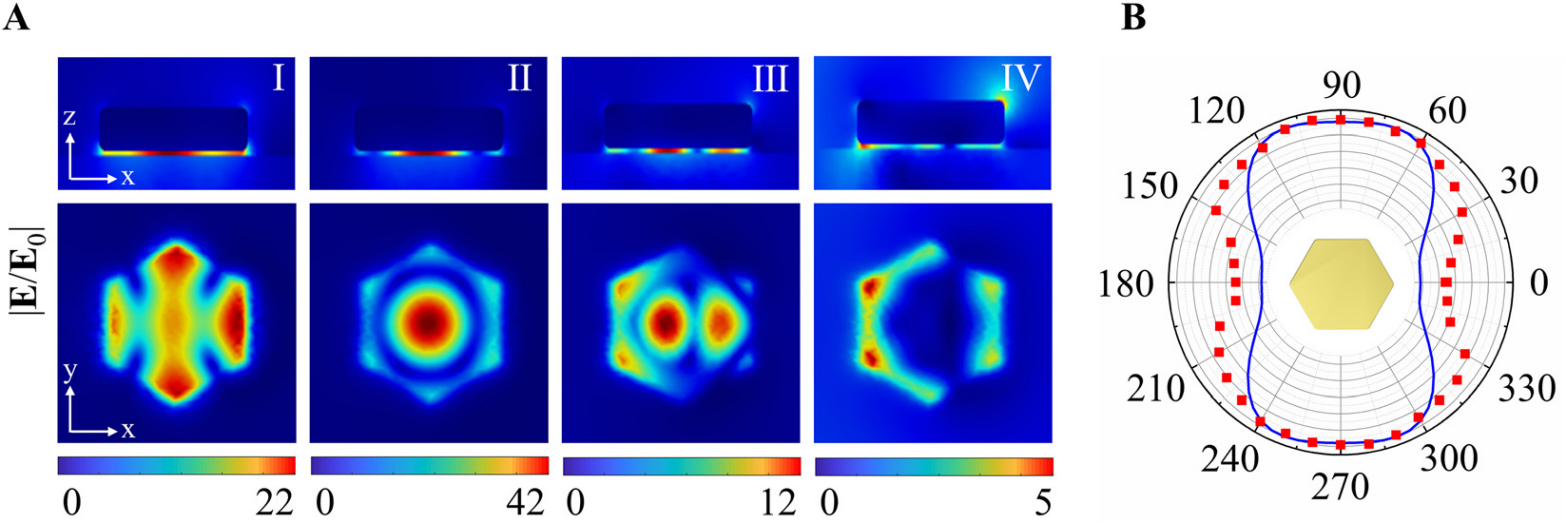
(A) Electric field enhancements of modes I–IV for a hexagonal Au NPoM with *d* = 185 nm and *g* = 5 nm. (B) Polar plot of the polarization-dependent scattering intensity of the fundamental radial breathing mode (RBM, mode II). The blue solid line and red dots are derived from the simulation and experiment, respectively.

We then turn to examine the near- and far-field properties of the RBM. Figure 3A shows the electric field enhancement (defined as |*E*|/|*E*_0_|) maps of NPoM when illuminated by the p-polarized beam. It is noted that the electric field distributions of modes I–IV are in concordance with their charge patterns. Particularly, the fundamental RBM concentrates most of the electric field at the center of the nanoplate. More surprisingly, this RBM shows a maximum electric field enhancement over 40, almost 2 times larger than the typical dark mode, i.e., quadruple mode. In Figure 3B, we display the polar plot of scattering intensity for the RBM as a function of the polarization direction. As seen, the polar plot of the polarization distribution displays an anisotropic pattern in contrast to that of bare nanodisk [14]. It is because the charge in the nanoplate bottom induces another RBM but with the opposite distribution in the Au film as shown in Figure S3. Thus, the far field radiation can only be attributed to the charge oscillation on the top surface of the nanoplate as a consequence of the radiation canceling between two out-of-phase RBMs in the gap region. Additionally, the retardation effect of the excitation makes the charge distribution on the top surface of nanoplate distorted to be more like an electric dipole (see Figure S3), rendering a dumbbell-shaped polar diagram. The simulation also verifies this measured polar diagram.

**Figure 4:**
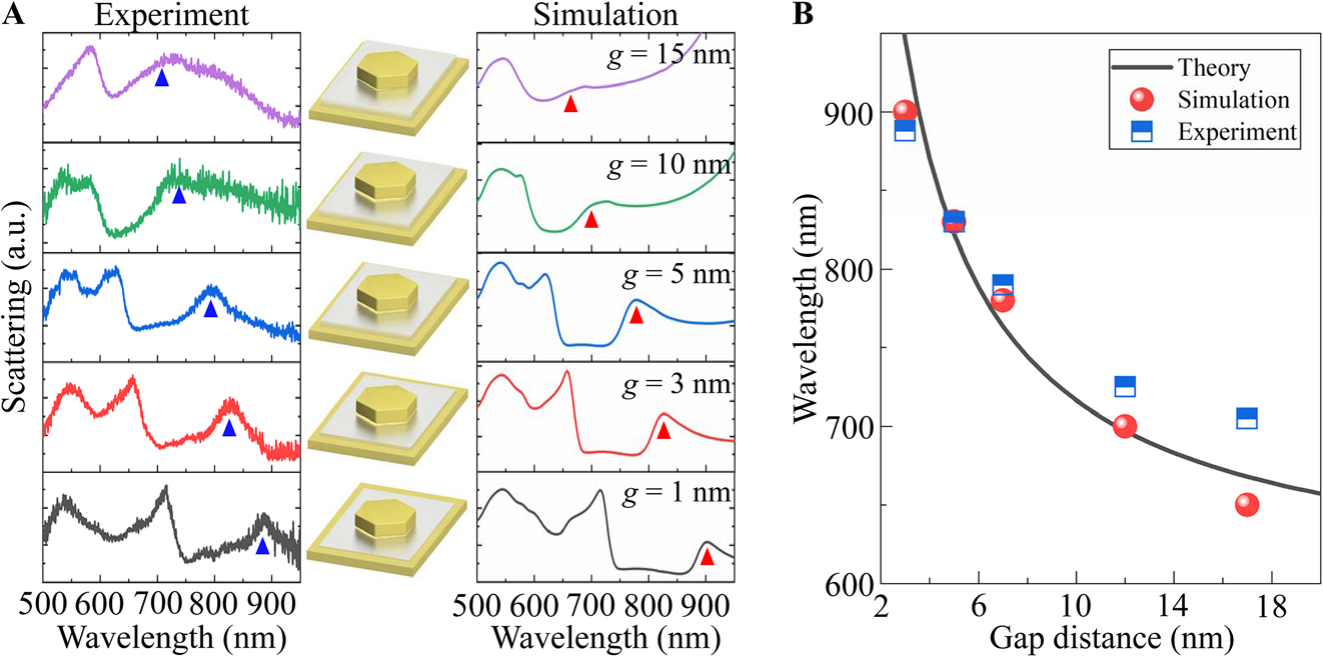
(A) Scattering spectra of hexagonal Au NPoMs (*d* = 180 nm) of various gap sizes. The left and right columns are the measured and simulated scattering spectra with triangles indicating the dispersion of RBM. The middle column depicts the NPoMs with different gap sizes. (B) Resonance wavelength of RBM as a function of gap size.

To further understand the nature of the RBM, we fabricate hexagonal Au NPoMs with different gap and nanoplate sizes, and focus our discussion on the variation of the resonance position for the fundamental RBM. From the measured scattering spectra shown in Figure 4A, we observe a blue shift of the RBM when the gap size of the hexagonal Au NPoM (*d* = 180 nm) is increased from 1 to 15 nm. This variation in experimental spectra is further confirmed by the simulation results in the right column of Figure 4A. Physically, for the RBM, charges with the opposite polarity accumulate at the bottom of Au nanoplate and the top of Au film, as a whole, form a bonding mode (see Supplementary Material, Figure S3). The frequency of this bonding mode can be generally described by $\omega^{2}\propto \omega_{1}^{2}-\kappa^{2}/\left(\omega_{2}^{2}-\omega_{1}^{2}\right)$ [44], where *ω*_1,2_ are eigenfrequencies of two independent modes in the Au nanoplate and the metal film, and *κ* is the coupling strength. As the increasing of the gap size, the coupling strength decreases, accompanied by the blue shift of breathing mode. For a quantitative interpretation, we compare the measured and simulated data with a circuit model [37, 45], [46], [47], [48] describing the film-coupled-particle system

(1) $$\lambda =\lambda_{p}\sqrt{2\epsilon_{m}+\epsilon_{\infty }+4\epsilon_{m}\eta }\text{.}$$

Here, *λ*_p_ = 148 nm denotes plasmon wavelength of Au, *ε*_m_ = 1 and *ε*_∞_ = 14 represent the permittivity of the dielectric medium and the corresponding background permittivity, respectively. *η* = *C*_g_/*C*_np_ is the capacitance of MIM waveguide $C_{\text{g}}=\epsilon_{m}\epsilon_{\text{g}}^{a}A/g$ normalized by that of nanoplate *C*_np_ = *bε*_m_π*R*, where *A* is the effective area with the radius of 40 nm determined by the net charge distribution of the fundamental RBM. *ε*_g_ = 2.4 represents the permittivity of Al_2_O_3_ and *R* = 94 nm is the equivalent radius representing the net charge area of the bottom surface of the nanoplate (*d* = 180 nm). For the hexagonal Au NPoM, the parameters *a* and *b* are 0.4 and 1.4, respectively. In Figure 4B, we show the dispersion of RBM against the gap size. It is noted that the circuit model is in accordance with both the simulation and experimental results, behaving the monotonously decreasing resonance wavelength on the gap size increasing.

**Figure 5:**
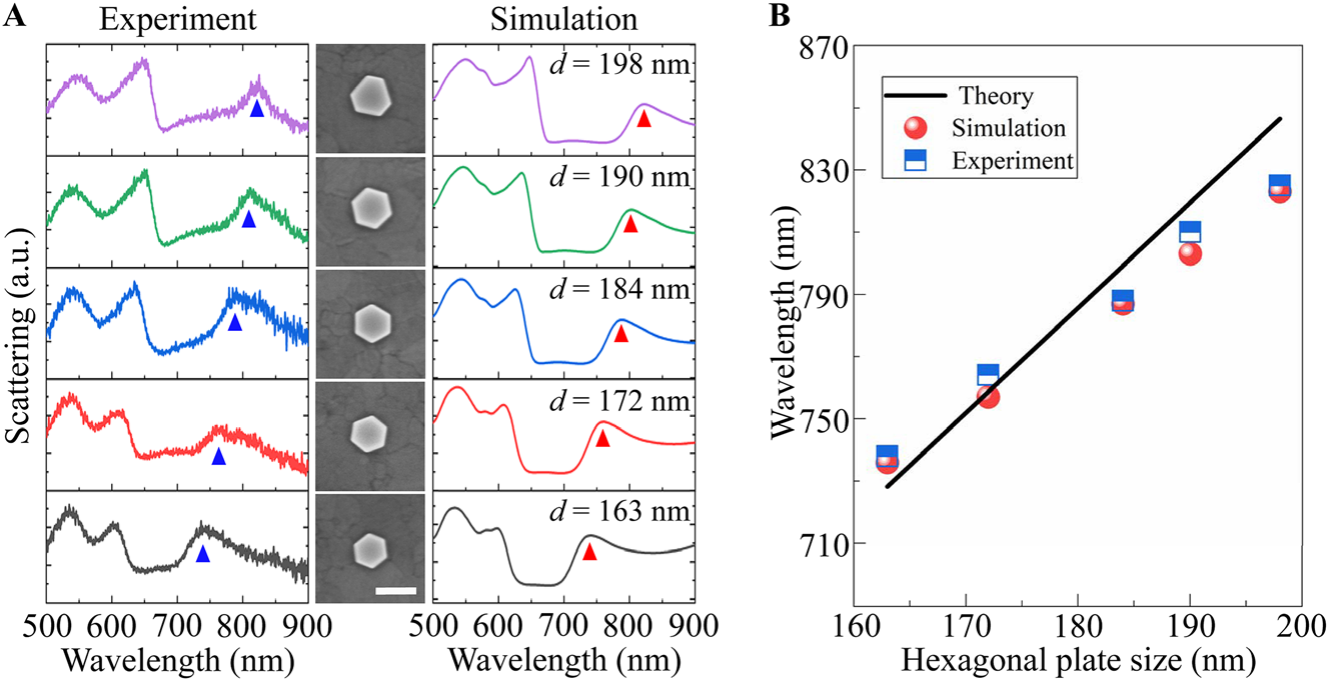
(A) Scattering spectra of hexagonal Au NPoM for the nanoplate size changing from 163–198 nm. The left and right columns show the experiment and simulation results. The triangle dots follow the spectral position of the RBM. The middle column displays the SEM images of the hexagonal Au nanoplate, where the scale bar is 200 nm. (B) Resonance wavelength of RBM versus nanoplate size. The blue square dots and red circle dots are for the results extracted from experiment and simulation spectra in (A). The black line represents the dispersion predicted by the Fabry–Pérot condition.

In Figure 5, we inspect the scattering spectra of hexagonal Au NPoM (*g* = 5 nm) as a dependence of the nanoplate size. Here, the sizes of nanoplates are measured from SEM images shown in the middle column of Figure 5A, which vary from 163 to 198 nm. It is found that the measured spectral peak of the RBM redshifts with increasing the nanoplate size (the left column of Figure 5A), which is also verified by the simulation results (the right column of Figure 5A). As mentioned, the RBM can be viewed as the standing plasmon waves in the nanoplate. Accordingly, we describe the dispersion of the RBM by the Fabry–Pérot condition [49, 50].

(2) $$\lambda =\frac{\pi dn_{\mathrm{eff}}}{a_{\mathrm{mn}}-\beta }\text{,}$$

where *λ*, *n*_eff_ and *β* are, respectively, the resonant wavelength, the effective index and the phase acquired by the reflection at the nanoplate boundary. For the RBM, *a*_mn_ is set to be *a*_01_ = 2.4, denoting the 1st root of the zeroth order Bessel function *J*_0_. The effective indices of the RBM for different nanoplate sizes are solved by the dispersion relation in Ref. [40, 49]. Figure 5B summarizes the wavelength of RBM as a function of the nanoplate size. The blue square and red circle dots depict the data retrieved from experimental and simulated spectra, respectively. As seen, the experiment agrees well with the simulation and both can be well described by a linear dispersion relation govern by Eq. (2) (black line in Figure 5B).

It is worth mentioning that the dark mode nature ensures the plasmonic RBM stores a large quantity of electromagnetic energy near the nanoplate, especially at the center. This is highly favorable to embed the active materials such as quantum dots, molecular dyes and two-dimensional semiconductors in the gap of the nanocavity to boost the absorption, emission and vibrational processes. Besides, the ease of tuning the wavelength of plasmonic RBM makes it facile to resonantly enhance these optical processes, facilitating the implement of high efficient optoelectronics devices including the photovoltaics cell, light emitting diode and nanolaser. On the other hand, as seen from Figure 3A, the RBM possesses a large surface-to-volume ratio of the local field enhancement, leading to a considerable electric field to penetrate in the surrounding media. As a result, the resonance peak of RBM is quite sensitive to the change of surrounding refractive index [51]. This merit working with the narrow spectral linewidth (see Figure 2A) could empower RBM with an excellent sensing performance.

## 4 Conclusions

In summary, plasmonic RBMs having a vanishing dipole moment are almost unattainable in the optical measurements. We have demonstrated an optical way to probe this dark mode in a plasmonic nanocavity composed of the closely spaced hexagonal Au nanoplate and ultrasmooth Au film. The polarization-resolved scattering measurements corroborated by the full-wave simulations allow us to uncover that the RBM arises from the standing plasmon waves confined by the nanoplate and becomes visible in the far-field due to the scattering by the nanoplate edges. Further, near field analysis shows that the RBM has a surprising ability of the electric field enhancement even beyond the typical dark modes such as quadrupole mode. Moreover, this RBM has a strong dispersion against the gap and nanoplate sizes, providing a readily way to tune its resonance wavelength across from visible to near-infrared region. Our findings provide an elegant way to excite the plasmonic RBM that could remove the obstacles for its applications in the field of nanophotonics.

## Supplementary Material

Supplementary Material
