# Supplementary material for: Unveiling radial breathing mode in a particle-on-mirror plasmonic nanocavity
Source: Nanophotonics. 2022 Jan 4;11(3):487–94. doi: 10.1515/nanoph-2021-0506 (PMC11501202; doi:10.1515/nanoph-2021-0506)
Supplement: Supplementary file 1 — Supplementary Material [file j_nanoph-2021-0506_suppl.docx]

**Supplementary Material for:**

**Unveiling Radial Breathing Mode in a Particle-on-Mirror Plasmonic Nanocavity**

Qifa Wang^1^, Chenyang Li^1^, Liping Hou^1^, Hanmou Zhang^1^, Xuetao Gan^1^, Kaihui Liu^2^, Malin Premaratne^3^, Fajun Xiao*^,1^ and Jianlin Zhao*^,1^

^1^Key Laboratory of Light Field Manipulation and Information Acquisition, Ministry of Industry and Information Technology, and Shaanxi Key Laboratory of Optical Information Technology, School of Physical Science and Technology, Northwestern Polytechnical University, Xi’an 710129, China

^2^State Key Laboratory for Mesoscopic Physics, Collaborative Innovation Centre of Quantum Matter, School of Physics, Peking University, Beijing 100871, China

^3^Advanced Computing and Simulation Laboratory (AχL), Department of Electrical and Computer Systems Engineering, Monash University, Clayton, Victoria 3800, Australia

*E-mail: [fjxiao@nwpu.edu.cn](mailto:fjxiao@nwpu.edu.cn)

*E-mail: [jlzhao@nwpu.edu.cn](mailto:jlzhao@nwpu.edu.cn)


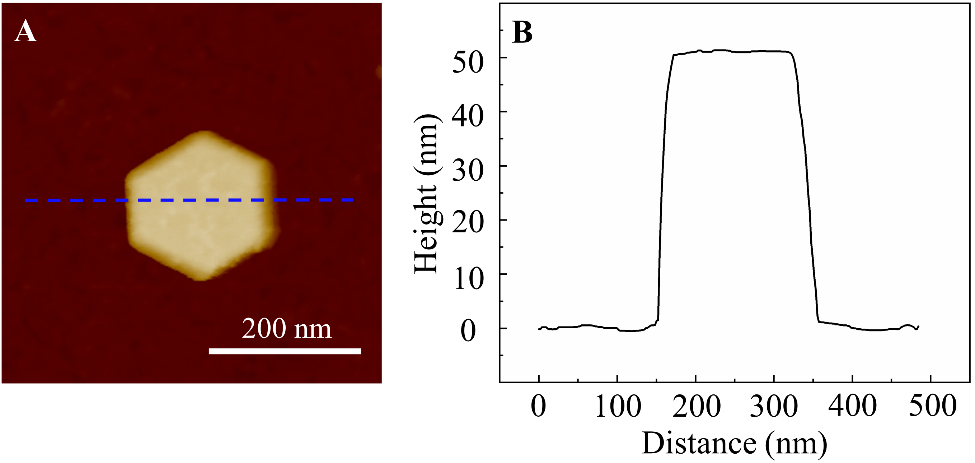


**Figure S1.** (A) AFM height image of an individual hexagonal Au NPoM. (B) Height profile extracted along the dashed line indicated in (A).


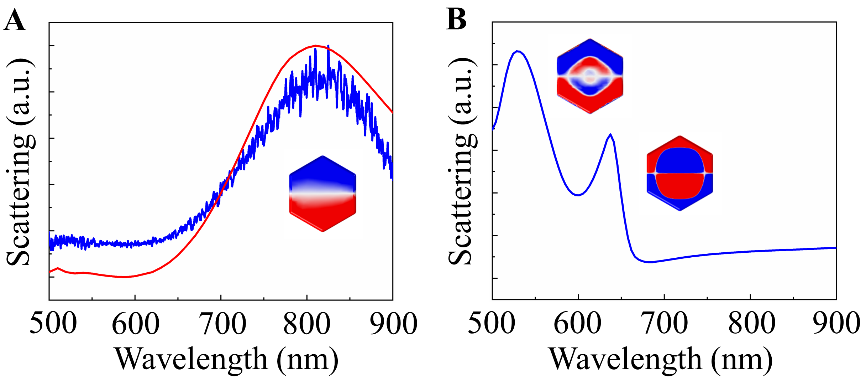


**Figure S2.** (A) Experimental (blue line) and simulated (red line) scattering spectra of hexagonal Au nanoplate (*d*=185 nm) on a silica substrate illuminated by p-polarized beam for an incidence angle of 70°. The inset shows the charge distribution of the only peak. (B) Simulated scattering spectra of hexagonal Au NPoM (*d*=185 nm, *g*=5 nm) illuminated by normal incidence beam. The inset depicts the charge distributions of the two peaks.


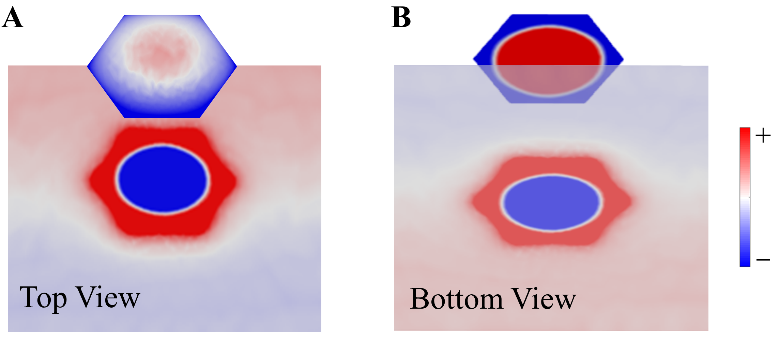


**Figure S3.** Surface charge density distributions of radial breathing mode are displayed with (A) 45° top view and (B) 45° bottom view.
